# SGLT2 inhibitors decrease cardiovascular death and heart failure hospitalizations in patients with heart failure: A systematic review and meta-analysis

**DOI:** 10.1016/j.eclinm.2021.100933

**Published:** 2021-06-05

**Authors:** Rhanderson Cardoso, Fabrissio P. Graffunder, Caique M.P. Ternes, Amanda Fernandes, Ana V. Rocha, Gilson Fernandes, Deepak L. Bhatt

**Affiliations:** a Heart and Vascular Center, Brigham and Women's Hospital, Harvard Medical School, Boston, MA, United States; b Division of Medicine, Federal University of Santa Catarina, Florianopolis, Brazil; c Cardiac Arrhythmia Service, SOS Cardio Hospital, Florianopolis, Brazil; d Division of Medicine, University of Miami, Miami, United States; e Division of Medicine, Federal University of Goias, Goiania, Brazil; f Division of Cardiology, University of Miami, Miami, United States

**Keywords:** Heart failure, Type 2 Diabetes, SGLT2 inhibitors, cardiovascular risk, DM, diabetes mellitus, eGFR, estimated glomerular filtration rate, HF, heart failure, HFpEF, heart failure with preserved ejection fraction, HR, hazard ratio, LVEF, left ventricular ejection fraction, NYHA, New York Heart Association, OR, odds ratio, RCTs, randomized controlled trials, SGLT2, sodium-glucose cotransporter 2

## Abstract

**Background:**

Sodium–glucose cotransporter 2 (SGLT2) inhibitors reduce the composite of heart failure (HF) hospitalizations or cardiovascular mortality among patients with HF. However, the efficacy of SGLT2 inhibitors in secondary endpoints of randomized trials and in subgroups of HF patients is not well known.

**Methods:**

We performed a systematic review and meta-analysis of placebo-controlled, randomized trials of SGLT2 inhibitors in patients with HF. PubMed, Embase, and Cochrane databases were searched for trials published up to January 21, 2021. Data were extracted from published reports and quality assessment was performed per Cochrane recommendations. Hazard ratios (HRs) with 95% CI were pooled across trials. The primary endpoints of interest were all-cause and cardiovascular mortality.

**Results:**

Out of 3969 database results, 15 randomized trials and 20,241 patients were included; 10,594 (52·3%) received SGLT2 inhibitors. All-cause mortality (HR 0·86; 95% CI 0·79–0·94; *p* = 0·0007; I^2^=0%) and cardiovascular mortality (HR 0·86; 95% CI 0·78–0·96; *p* = 0·006; I^2^=0%) were significantly lower in patients treated with SGLT2 inhibitors compared with placebo. The composite of cardiovascular mortality, HF hospitalizations, or urgent visits for HF was significantly reduced with SGLT2 inhibitors in all the following subgroups: male, female, age < 65, age ≥ 65, race – Black and White, estimated glomerular filtration rate (eGFR) <60, eGFR ≥60, New York Heart Association (NYHA) class II, NYHA ≥III, and HF with preserved ejection fraction.

**Interpretation:**

In patients with HF, SGLT2 inhibitors significantly reduce all-cause and cardiovascular mortality compared with placebo. In addition, the composite of cardiovascular mortality or HF hospitalizations/urgent visits is reduced with SGLT2 inhibitors across subgroups of sex, age, race, eGFR, HF functional class, and ejection fraction.

**Research in context**

**Evidence before this study**

Sodium-glucose cotransporter-2 (SGLT2) inhibitors have been shown to reduce the composite of cardiovascular mortality and heart failure hospitalizations among patients with heart failure in multiple cardiovascular outcome trials. However, there have been conflicting results with regards to the effect of SGLT2 inhibitors in mortality endpoints among patients with HF, possibly due to a lack of power for secondary trial endpoints. Therefore, we aimed to perform a systematic review and meta-analysis of randomized, placebo-controlled trials of SGLT2 inhibitors in patients with HF, specifically interested in mortality endpoints. We searched PubMed, Embase, and Cochrane on January 21, 2021 using the following medical subject heading terms: ‘heart failure’, ‘SGLT2’, ‘sodium-glucose co-transporter-2′, ‘canagliflozin’, ‘dapagliflozin’, ‘empagliflozin’, ‘sotagliflozin’, and ‘ertugliflozin’.

**Added value of this study**

This meta-analysis of 15 trials and over 20,000 patients found that SGLT2 inhibitors significantly reduce all-cause mortality, cardiovascular mortality, and HF hospitalizations among individuals with HF. In addition, the composite of cardiovascular death or HF hospitalizations/urgent visits was significantly reduced in patients treated with SGLT2 inhibitors stratified by age, sex, race, renal function, HF functional classification, ejection fraction, and in those with or without diabetes.

**Implications of all the available evidence**

The reduction in mortality and hospitalization endpoints indicates that SGLT2 inhibitors should be considered as part of standard care in patients with HF. Further studies are warranted to evaluate the efficacy of SGLT2 inhibitors in subgroups of patients with HF, particularly in those with HF and preserved ejection fraction.

Alt-text: Unlabelled box

## 1 Introduction

Diabetes mellitus (DM) is a well-established risk factor for cardiovascular diseases, including heart failure (HF)[1], [2], [3], [4], [5]. Approximately 1 in 7 individuals with DM and cardiovascular disease have HF as the initial cardiovascular presentation, and many more go on to have HF associated with atherosclerotic syndromes [1]. Until recently, there were no HF therapies directed at glucose metabolism [2,3]. Although there is still an unmet need for additional HF therapies in patients with DM, sodium glucose co-transporter 2 (SGLT2) inhibitors have begun to change this paradigm [6,7]. SGLT2 are major transport proteins responsible for reabsorption of glucose in the kidneys. Landmark cardiovascular outcome trials have shown a benefit of SGLT2 inhibitors over placebo in the composite endpoint of cardiovascular mortality or HF hospitalizations [8], [9], [10], [11], [12], [13], [14], [15].

Despite multiple studies, the efficacy of SGLT2 inhibitors on individual (non-composite) endpoints, such as all-cause mortality and cardiovascular mortality, is not clear. These important clinical outcomes are often studied as secondary endpoints in the individual randomized trials. Therefore, trials typically lack enough power for a definitive assessment of such endpoints, especially in subgroups. Indeed, most trials of SGLT2 inhibitors including patients with DM, HF, or both, have shown no significant benefit in all-cause or cardiovascular mortality [10], [11], [12], [13], [14], [15], [16], [17]. Similarly, individual trials lack enough power to detect significant outcome differences in population subgroups, such as those defined by age, sex, race, renal function, and left ventricular ejection fraction (LVEF). Therefore, we sought to perform a systematic review and meta-analysis examining the efficacy of SGLT2 inhibitors in patients with HF, with or without diabetes, specifically interested in mortality and hospitalization endpoints, as well as the outcomes in subpopulations of HF patients.

## 2 Methods

### 2.1 Search strategy and selection criteria

The systematic review and meta-analysis were performed in line with recommendations from the Cochrane Collaboration and the Preferred Reporting Items for Systematic Reviews and Meta-Analysis (PRISMA) statement guidelines [18]. The pre-specified research protocol was not published. We systematically searched Embase, Cochrane Central Register of Controlled Trials, and PubMed from inception to January 21, 2021 for studies published in English with the following medical subject heading terms: ‘heart failure’, ‘SGLT2’, ‘sodium-glucose co-transporter-2’, ‘canagliflozin’, ‘dapagliflozin’, ‘empagliflozin’, ‘sotagliflozin’, and ‘ertugliflozin’. In addition, the references of included studies and systematic reviews were evaluated for additional studies. A complete electronic search strategy is reported in the Supplementary Appendix.

We included studies that met the following eligibility criteria: (1) randomized controlled trials (RCTs); (2) comparing SGLT2 inhibitors with placebo; (3) in patients with HF or in a subgroup of HF patients within the trial; and (4) reporting at least one of the clinical outcomes of interest. We excluded studies with (1) overlapping patient populations; (2) without a placebo control group; or (3) with a crossover design. Randomized trials of SGLT2 inhibitors in patients with and without HF were included only if they reported dedicated outcomes in the HF population.

Cardiovascular outcome trials are typically powered for a composite of major adverse cardiac events, lacking enough power to evaluate statistical significance of secondary, yet clinically relevant endpoints. Therefore, we sought to perform a systematic review and meta-analysis of these endpoints. We extracted data for: (1) all-cause mortality; (2) cardiovascular mortality; and (3) hospitalizations for HF. These three outcomes were compared using pooled hazard ratios (HR) to preserve time-to-event data from individual studies. We also performed a meta-analysis of (1) urgent HF visits; (2) amputations; (3) fractures; and (4) weight change.

Importantly, we sought to evaluate the efficacy of SGLT2 inhibitors relative to placebo in subgroups of HF patients. Specifically, we performed pre-specified analyses of the composite of cardiovascular mortality or HF hospitalizations/urgent visits in the following subgroups: (1) male; (2) female; (3) age ≥65 years old; (4) age <65 years old; (5) White race; (6) Black race; (7) estimated glomerular filtration rate (eGFR) ≥60 mL/min/1·73 m^2^; (8) eGFR <60 mL/min/1·73 m^2^; (9) New York Heart Association (NYHA) functional class II; (10) NYHA III or IV; (11) HF with reduced LVEF; and (12) heart failure with preserved ejection fraction (HFpEF). Criteria for preserved LVEF differed slightly between studies, ranging from ≥45% to ≥50%. In addition, we performed a post-hoc analysis in patients with DM and without DM.

### 2.2 Data analysis

Two authors (F.G. and C.T.) independently extracted baseline characteristics reported in Table 1 and outcomes data using prespecified criteria for search, data extraction, and quality assessment. Disagreements were resolved by consensus among three authors (R.C., F.G., and C.T.). Treatment effects for binary endpoints were compared using pooled HR or odds-ratios (OR) with 95% confidence intervals. As described, mortality and HF hospitalization outcomes were analyzed with HR to preserve time-to-event data. Weighted mean differences were used to pool continuous outcomes. Heterogeneity was evaluated with Cochran Q test and I^2^ statistics; p values inferior to 0·10 and I^2^>25% were considered significant for heterogeneity. We used a fixed-effect model for endpoints with I^2^ < 25% (low heterogeneity). In pooled outcomes with high heterogeneity, DerSimonian and Laird random-effects model was used. Review Manager 5·4 (Nordic Cochrane centre, The Cochrane Collaboration, Copenhagen, Denmark) was used for statistical analysis. We used the Cochrane Collaboration's tool for assessing risk of bias in randomized trials for quality assessment of individual randomized studies [19]. Each trial received a score of high, low, or unclear risk of bias in 5 domains: selection, performance, detection, attrition, and reporting biases. Funnel plots of study weights vs. point estimates were used to assess for evidence of publication bias.

**Table 1:** Baseline characteristics of included studies.

	Number of HF patients	SGLT2 inhibitor	Female, n(%)	Mean/ median age (years)	Black, n(%)	HFpEF, n(%) LVEF criteria	Diabetes, n(%)	Mean/median eGFR (mL/min/1·73m^2^)	NYHA II, n(%)	NYHA III/IV, n(%)	Follow-up (months)
**de Boer** 2020 [29]	63	empagliflozin	24 (38·1)	70	1 (1·6)	48 (76·2) LVEF ≥45%	63 (100)	NA	47 (74·6)	16 (25·4)	3
**CANVAS HF** 2018 [9,28]	1461	canagliflozin	648 (44·3)	63·8	28 (1·9)	NA	1461 (100)	73·0	NA	NA	47
**CREDENCE** 2019 [26,27]	652	canagliflozin	257 (39·4)	65·2	27 (4·1)	NA	652 (100)	57·0	359 (55·1)	70 (10·7)	31·4
**DAPA-HF** 2019 [8,30]	4744	dapagliflozin	1109 (23·3)	66·3	226 (4·7)	0	1983 (41·8)	65·7	3203 (67·5)	1541 (32·5)	18·2
**DECLARE- TIMI 58** 2019 [10,17]	1724	dapagliflozin	37·4%*	63·9*	3·5%*	808 (46·9) LVEF ≥45%	1724 (100)	85·2*	1114 (64·6)	154 (8·9)	50·4
**DEFINE-HF** 2019 [24]	263	dapagliflozin	70 (26·6)	61·3	99 (37·6)	0	166 (63·1)	69	173 (65·6)	90 (34·2)	3
**EMPA-REG OUTCOME** 2015 [11,25]	706	empagliflozin	211 (29·9)	64·5	357 (5)*	NA	706 (100)	65·2	NA	NA	37·2
**EMPA-TROPISM** 2020 [23]	84	empagliflozin	30 (35·7)	62	16 (19)	0	0	81·5	NA	NA	6
**EMPEROR-Reduced** 2020 [12]	3730	empagliflozin	893 (23·9)	66·8	257 (6·9)	0	1856 (49·7)	62	2800 (75)	930 (24·9)	16
**EMPIRE HF** 2020 [22]	190	empagliflozin	28 (14·7)	64	NA	0	24 (12·6)	74	149 (78·4)	29 (15·2)	3
**REFORM** 2020 [21]	56	dapagliflozin	19 (33·9)	67·1	NA	NA	56 (100)	72	24 (42·9)	7(12·5)	12
**SCORED** 2020 [13]	3283	sotagliflozin	44·9%*	69*	364 (3·4)*	1667 (50·7) LVEF ≥50%	3283 (100)	44·5*	NA	NA	16
**SOLOIST-WHF** 2020 [14]	1222	sotagliflozin	412 (33·7)	70	50 (4)	256 (20·9) LVEF ≥50%	1222 (100)	49·7	552 (45·1)	614 (50·2)	9
**SUGAR-DM-HF** 2020 [20]	105	empagliflozin	28 (26·6)	68·7	NA	0	82 (78·1)	67·3	81 (77·1)	24 (22·9)	9
**VERTIS** 2020 [15,16]	1958	ertugliflozin	624 (31·8)	64·4*	235 (2·8)*	1007 (51·4) LVEF >45%	1958 (100)	76*	1289 (95·8)	140 (7·1)	42

*Data in the entire study population, not just in patients with heart failure; eGFR: estimated glomerular filtration rate; HF: heart failure; HFpEF: heart failure with preserved ejection fraction; LVEF: left ventricular ejection fraction; NA: not available; NYHA: New York Heart Association functional classification; SGLT2i: sodium-glucose co-transporter 2.

### 2.3 Role of the funding source

There was no funding source for this study. All authors had full access to all the data in the study. The corresponding author had final responsibility for the decision to submit for publication.

## 3 Results

**Fig. 1:**
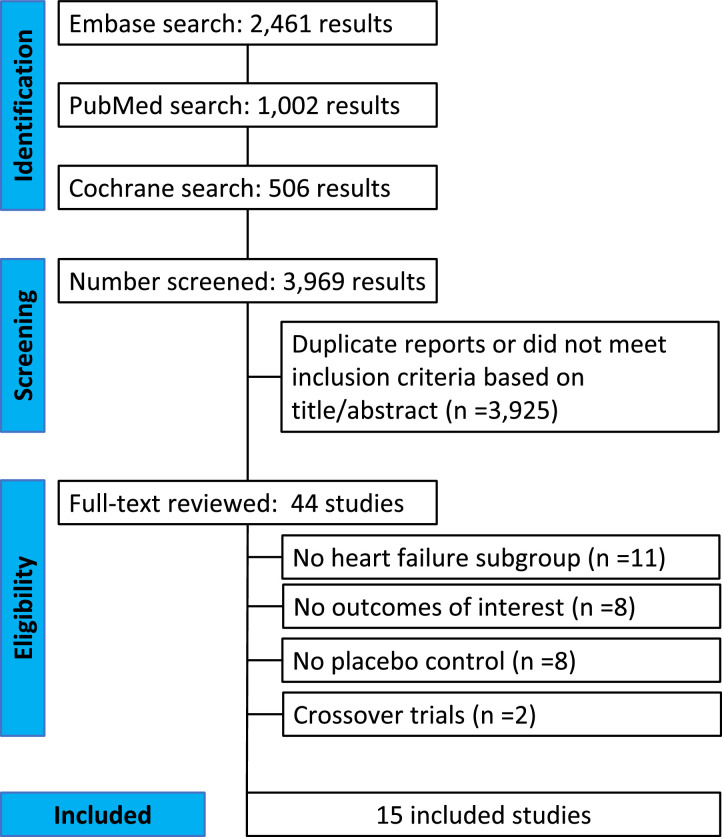
PRISMA flow diagram of study screening and selection. The search strategy in Embase, PubMed, and Cochrane yielded 3969 studies, of which 44 were fully reviewed for inclusion and exclusion criteria. A total of 15 studies were included in the meta-analysis.

As detailed in Fig. 1, the initial search yielded 3969 results. After removal of duplicate records and studies with an exclusion criterion based on title/abstract review, 44 remained and were fully reviewed for the inclusion and exclusion criteria. Ultimately, a total of 20,241 patients from 15 RCTs were included in this systematic review and meta-analysis [[8], [9], [10], [11], [12], [13], [14], [15], [16], [17],[20], [21], [22], [23], [24], [25], [26], [27], [28], [29], [30]]. SGLT2 inhibitors were prescribed in 10,594 (52·3%) patients. Study characteristics are reported in Table 1. A total of 3384 (31·9%) patients received dapagliflozin, 2544 (24·0%) received empagliflozin, 2248 (21·2%) received sotagliflozin, 1286 (12·1%) received ertugliflozin and 1132 (10·7%) received canagliflozin. Four studies with a total of 3738 patients reported dedicated outcomes in patients with HFpEF, with LVEF cutoffs ranging from ≥45% to ≥50%. Mean follow-up ranged from 3 to 50·4 months.

**Fig. 2:**
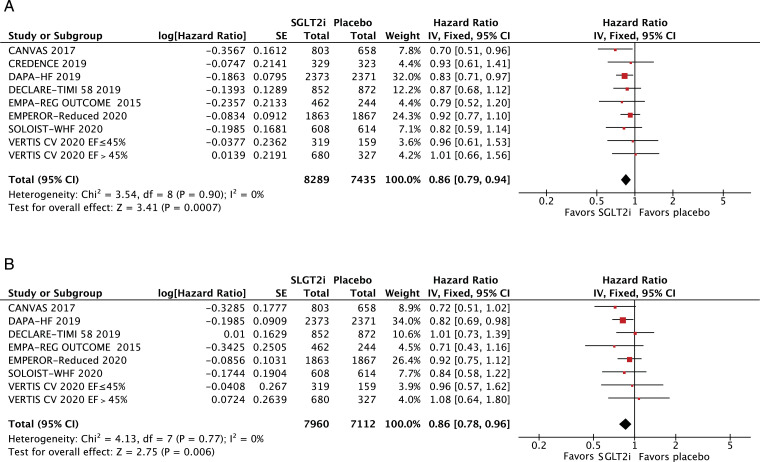
A. Title: All-cause mortality was significantly lower in the SGLT2 inhibitor group. Legend: There was a significant 14% relative risk reduction in all-cause mortality among patients with HF treated with SGLT2 inhibitors compared with placebo (OR 0•86; 95% CI 0•79–0•94). B. Title: Cardiovascular mortality was significantly lower in the SGLT2 inhibitor group. Legend: There was a significant 14% relative risk reduction in cardiovascular mortality among patients with HF treated with SGLT2 inhibitors compared with placebo (OR 0•86; 95% CI 0•78–0•96).

**Fig. 3:**
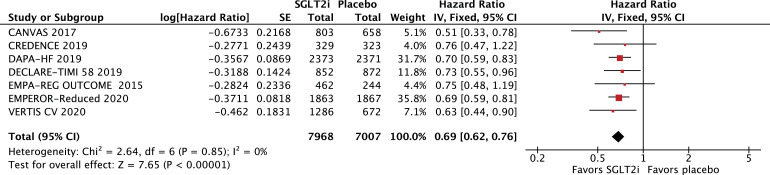
Title: Heart failure hospitalizations were significantly lower in the SGLT2 inhibitor group. Legend: There was a significant 31% relative risk reduction in heart failure hospitalizations among patients with HF treated with SGLT2 inhibitors compared with placebo (OR 0•69; 95% CI 0•62–0•76).

**Fig. 4:**
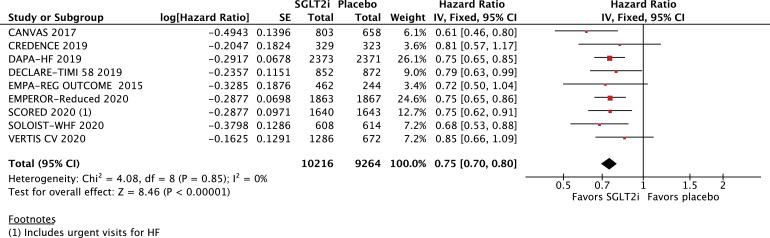
Title: Cardiovascular mortality or hospitalizations for HF was significantly lower in the SGLT2 inhibitor group. Legend: There was a significant 25% relative risk reduction in the composite endpoint of cardiovascular mortality or heart failure hospitalizations among patients with HF treated with SGLT2 inhibitors compared with placebo (OR 0•75; 95% CI 0•70–0•80).

All-cause mortality (HR 0·86; 95% CI 0·79–0·94; *p* = 0·0007; I^2^=0%; Fig. 2A) and cardiovascular mortality (HR 0·86; 95% CI 0·78–0·96; *p* = 0·006; I^2^=0%; Fig. 2B) were significantly lower among patients treated with SGLT2 inhibitors. In addition, hospitalizations for HF (HR 0·69; 95% CI 0·62–0·76; *p*<0·0001; I^2^=0%; Fig. 3) and urgent visits for HF (OR 0·39; 95% CI 0·22–0·69; *p* = 0·001; I^2^=0%; Supplementary Figure 1) were significantly reduced in those receiving SGLT2 inhibitors. The composite of cardiovascular mortality or hospitalizations for HF was also reduced in patients randomized to SGLT2 inhibitors (HR 0·75; 95% CI 0·70–0·80; *p*<0·0001; I^2^=0%; Fig. 4).

There was no significant difference between groups in terms of amputations (OR 1·39; 95% CI 0·86–2·24; *p* = 0·18; I^2^=0%; Supplementary Figure 2A) or bone fractures (OR 1·09; 95% CI 0·85–1·40; *p* = 0·51; I^2^=0%; Supplementary Figure 2B). Weight loss was significantly greater in those receiving SGLT2 inhibitors compared with placebo (mean difference −1·11 kg; 95% CI −1·41 to −0·82; *p*<0·0001; I^2^= 63%; Supplementary Figure 3).

**Fig. 5:**
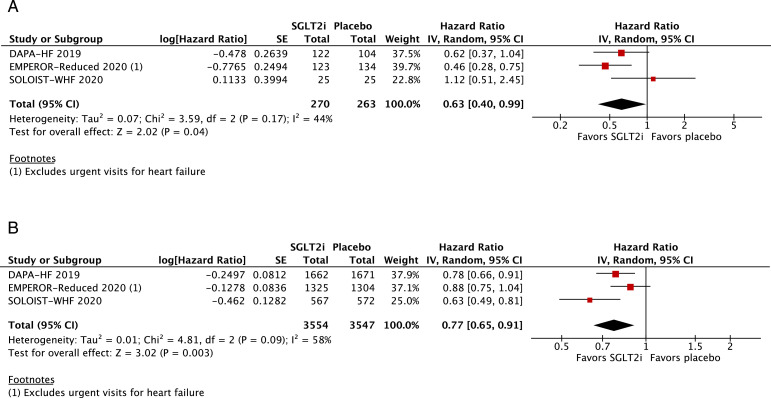
A. Title: Among Black patients, cardiovascular death or HF hospitalizations/urgent visits was significantly lower in the SGLT2 inhibitor group.Legend: In patients with HF who were Black, there was a significant 37% relative risk reduction in the composite of cardiovascular death or HF hospitalizations/urgent visits among those treated with SGLT2 inhibitors compared with placebo (OR 0•63; 95% CI 0•40–0•99). B. Title: Among White patients, cardiovascular death or HF hospitalizations/urgent visits was significantly lower in the SGLT2 inhibitor group. Legend: In patients with HF who were White, there was a 23% relative risk reduction in the composite of cardiovascular death or HF hospitalizations/urgent visits among those treated with SGLT2 inhibitors compared with placebo (OR 0•77; 95% CI 0•65–0•91).

**Fig. 6:**
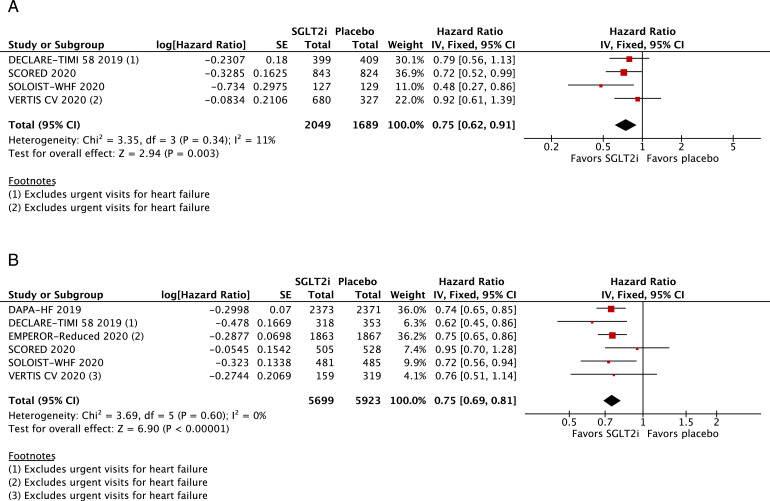
A. Title: Cardiovascular death or HF hospitalizations/urgent visits in the subgroup of HF with preserved ejection fraction. Legend: In the subgroup of HF with preserved ejection fraction, there was a 25% relative risk reduction in the composite endpoint of cardiovascular death or HF hospitalizations/urgent visits among those treated with SGLT2 inhibitors compared with placebo (OR 0•75; 95% CI 0•62–0•91). B. Title: Cardiovascular death or HF hospitalizations/urgent visits in the subgroup of HF with reduced ejection fraction. Legend: In the subgroup of HF with reduced ejection fraction, there was a 25% relative risk reduction in the composite endpoint of cardiovascular death or HF hospitalizations/urgent visits among those treated with SGLT2 inhibitors compared with placebo (OR 0•75; 95% CI 0•69–0•81).

We examined the composite of cardiovascular mortality, HF hospitalizations, or urgent visits for HF among selected HF subgroups. There was a consistent reduction in the composite outcome among the following groups of patients: men (HR 0·74; 95% CI 0·67–0·82; *p*<0·0001; I^2^=20%; Supplementary Figure 4A; *n* = 7282), women (HR 0·71; 95% CI 0·58–0·85; *p* = 0·0004; I^2^=9%; Supplementary Figure 4B; *n* = 2414), age <65 (HR 0·75; 95% CI 0·65–0·87; *p* = 0·0001; I^2^=0%; Supplementary Figure 5A; *n* = 3809), age ≥65 (HR 0·73; 95% CI 0·65–0·81; *p*<0·0001; I^2^=0%; Supplementary Figure 5B; *n* = 5029), Black (HR 0·63; 95% CI 0·40–0·99; *p* = 0·04; I^2^=44%; Fig. 5A; *n* = 533), White (HR 0·77; 95% CI 0·65–0·91; *p* = 0·003; I^2^=58%; Fig. 5B; *n* = 7101), eGFR <60 (HR 0·74; 95% CI 0·67–0·82; *p*<0·0001; I^2^=23%; Supplementary Figure 6A; *n* = 6954), eGFR ≥60 (HR 0·74; 95% CI 0·64–0·84; *p*<0·0001; I^2^=0%; Supplementary Figure 6B; *n* = 4746), NYHA class II (HR 0·66; 95% CI 0·58–0·74; *p*<0·0001; I^2^=0%; Supplementary Figure 7A; *n* = 6555), NYHA class ≥III (HR 0·86; 95% CI 0·76–0·99; *p* = 0·03; I^2^=0%; Supplementary Figure 7B; *n* = 3085), HFpEF (HR 0·75; 95% CI 0·62–0·91; *p* = 0·003; I^2^=11%; Fig. 6A; *n* = 3738) and HF with reduced EF (HR 0·75; 95% CI 0·69–0·81; *p*<0·0001; I^2^=0%; Fig. 6B; *n* = 11,622). In addition, the composite of cardiovascular death or HF hospitalizations was significantly lower in the SGLT2 inhibitor group among participants with diabetes (HR 0·74; 95% CI 0·68–0·80; *p*<0·0001; I^2^=0%; Supplementary Figure 8A) and without diabetes (HR 0·74; 95% CI 0·63–0·86; *p* = 0·0002; I^2^=0%; Supplementary Figure 8B).

Supplementary Table 1 outlines individual appraisal of each RCT included in the meta-analysis. Overall, all studies were deemed at low risk of bias. There was also no evidence of publication bias by funnel plots. There was a symmetrical distribution of studies with similar weights around the meta-analysis point estimate (Supplementary Figure 9A and 9B).

## 4 Discussion

In this systematic review and meta-analysis of 15 studies and 20,241 patients, we compared SGLT2 inhibitors with placebo in patients with HF. The main findings were as follows: (1) SGLT2 inhibitors were associated with a 14% relative reduction in the risk of all-cause mortality and cardiovascular mortality; (2) SGLT2 inhibitors reduced the risk of HF hospitalizations by 31%; (3) the composite of cardiovascular mortality, HF hospitalizations, or HF urgent visits was significantly lower in patients treated with SGLT2 inhibitors across subgroups of age, sex, race, renal function, and HF functional classification; and (4) in more than 3700 patients with HFpEF, the composite of cardiovascular mortality, HF hospitalizations, or HF urgent visits was reduced by 25% among patients treated with SGLT2 inhibitors.

The mechanisms by which DM can mediate the onset or worsening of HF are several-fold, including ischemia from epicardial coronary artery disease; microvascular dysfunction; myocyte hypertrophy; impaired mitochondrial function; dysautonomia; increased proinflammatory cytokines; and sodium retention due to up-regulation of sodium-glucose co-transporters [[2], [3], [4],^31^]. Along with co-morbidities such as obesity, hypertension, and kidney disease, these effects can lead to subclinical myocardial dysfunction or HF with reduced or preserved ejection fraction.^1,3,4^ Until the development of SGLT2 inhibitors, glucose-controlling therapies for DM had a neutral or harmful effect in HF endpoints [32], [33], [34], [35], [36].

SGLT2 inhibition opposes some of the adverse effects of DM and insulin resistance on cardiovascular metabolism and function, improving oxygen delivery, cardiac fuel energetics, and mitochondrial function [37], [38], [39], [40]. In addition, there are other positive effects of SGLT2 inhibitors on HF hemodynamics that may be independent of DM, such as natriuresis and preload reduction; beneficial effects on circulating provascular progenitor cells; blood pressure lowering and afterload reduction; and regression of left ventricular hypertrophy [41], [42], [43]. Renal protection and weight loss may also contribute to improved HF outcomes with SGLT2 inhibitors [44,45].

Initial trials of SGLT2 inhibitors in patients with DM were originally designed to demonstrate the cardiovascular safety of these agents, as mandated by the Food and Drug Administration [9,25]. Evidence of cardiovascular benefit with these agents led to the undertaking of large cardiovascular outcome trials, which have consistently shown a reduction in the composite of cardiovascular mortality or HF hospitalizations with SGLT2 inhibitors in patients with diabetes [8], [9], [10], [11], [12], [13], [14], [15]. However, whether there are heterogeneous treatment responses to SGLT2 inhibitors in subgroups of patients with HF is not well known. There are important nuances in HF physiology and treatment responses based on sex and race, for instance. [46], [47], [48] Moreover, many subgroups of patients, such as women and older patients, tend to be underrepresented in clinical trials. Therefore, it is usually not possible to draw firm conclusions about the outcomes of these subgroups in individual trials. In our meta-analysis, there was a similar 25–30% relative risk reduction in the composite outcome of cardiovascular death or HF hospitalizations/urgent visits in women (*n* = 2414) and men (*n* = 7282) treated with SGLT2 inhibitors. Patients randomized to SGLT2 inhibitors who self-identified as Black had a significant reduction in the same composite endpoint (HR 0·63; 95% CI 0·40–0·99; *n* = 533), as did patients who self-identified as White (HR 0·77; 95% CI 0·65–0·91; *n* = 7101).

Another HF population that requires a dedicated analysis is HFpEF. To date, proven therapies in HF with reduced ejection fraction have had disappointing, negative results in studies of HFpEF [49], [50], [51], [52], [53]. The previously mentioned cardiometabolic effects of SGLT2 inhibitors, such as improvement in cardiomyocyte energetics and natriuresis, are desirable in patients with HPpEF. Treatment with dapagliflozin in a mouse model of HFpEF led to improvements in global longitudinal strain and cardiac fibrosis [54]. In our meta-analysis, the pooled results of 4 studies and 3738 patients with HFpEF found a 25% relative risk reduction in the composite outcome of cardiovascular death or HF hospitalizations/urgent visits among those randomized to SGLT2 inhibitors. Two ongoing randomized trials are examining the efficacy of SGLT2 inhibitors in patients with HFpEF: Dapagliflozin Evaluation to Improve the Lives of Patients with Preserved Ejection Fraction Heart Failure (DELIVER; NCT03619213) and Empagliflozin Outcome Trial in Patients with Chronic Heart Failure with Preserved Ejection Fraction (EMPEROR-Preserved; NCT03057951).

Our study has limitations. First, the absence of patient-level data precluded a more complete report on the HF subpopulations. The only outcome studied for the subgroup analyses was the composite of cardiovascular death and HF hospitalizations or urgent visits. Although we planned to analyze all-cause and cardiovascular mortality in subgroups of HF patients, this was not possible because studies most commonly reported subgroup analyses only for their primary endpoint, the composite of cardiovascular mortality and HF hospitalizations. Also, there was substantial variability in the average follow-up period between studies, ranging from 3 to 50 months. However, the heterogeneity of pooled outcomes was quite low for most outcomes, which corroborates prior findings of an early onset of the benefit of SGLT2 inhibitors [55]. Also, the absence of patient-level data did not allow for the reporting of a pooled number of events. Instead, hazards ratios were computed and reported. Finally, we pooled the outcomes of all SGLT2 inhibitors under the same intervention group. Whether there are differences in HF outcomes between different drugs, particularly given the dual inhibition of SGLT2 and SGLT1 by sotagliflozin, could not be assessed in our study.

In conclusion, SGLT2 inhibitors reduce all-cause mortality, cardiovascular mortality, and HF hospitalizations in patients with HF. The composite of cardiovascular death or HF hospitalizations/urgent visits are consistently reduced by SGLT2 inhibitors across multiple subgroups, including women, older patients, Black individuals, and those with impaired renal function. Finally, the composite of cardiovascular mortality or HF hospitalizations/urgent visits appears to be significantly reduced by SGLT2 inhibitors in patients with HFpEF. These findings support the use of SGLT2 inhibitors as a new pillar of HF therapy.

## Funding

There was no funding source for this study.

## Contributors

**RC:** conceptualization, study design, data analysis, data interpretation, writing (original draft), writing (review and editing)

**FPG:** data collection, data analysis, data interpretation, writing (original draft)

**CMPT:** data collection, data analysis, data interpretation, writing (original draft)

**AF:** conceptualization, study design, writing (review and editing)

**AVR:** data collection, data analysis, data interpretation

**GF:** conceptualization, study design, writing (review and editing)

**DLB:** conceptualization, data interpretation, writing (review and editing)

## Data sharing agreement

Because this meta-analysis was based on data extracted from previously published research, all of the data and study materials are available in the public domain. The authors of this meta-analysis do not have access to patient-level data of the individual studies. Researchers with an interest in individual-level data from the studies included in this meta-analysis are encouraged to contact the corresponding author from each individual study for such requests.

## Declaration of Interests

Dr. Bhatt reports grants from Amarin, grants from AstraZeneca, grants from Bristol-Myers Squibb, grants from Eisai, grants from Ethicon, grants from Medtronic, grants from sanofi aventis, grants from The Medicines Company, other from FlowCo, grants and other from PLx Pharma, other from Takeda, personal fees from Duke Clinical Research Institute, personal fees from Mayo Clinic, personal fees from Population Health Research Institute, personal fees, non-financial support and other from American College of Cardiology, personal fees from Belvoir Publications, personal fees from Slack Publications, personal fees from WebMD, personal fees from Elsevier, other from Medscape Cardiology, other from Regado Biosciences, other from Boston VA Research Institute, personal fees and non-financial support from Society of Cardiovascular Patient Care, non-financial support from American Heart Association, personal fees from HMP Global, grants from Roche, personal fees from Harvard Clinical Research Institute (now Baim Institute for Clinical Research), other from Clinical Cardiology, personal fees from Journal of the American College of Cardiology, other from VA, grants from Pfizer, grants from Forest Laboratories/AstraZeneca, grants from Ischemix, other from St. Jude Medical (now Abbott), other from Biotronik, grants and other from Cardax, other from Boston Scientific, grants from Amgen, grants from Lilly, grants from Chiesi, grants from Ironwood, personal fees from Cleveland Clinic, personal fees from Mount Sinai School of Medicine, other from Merck, grants from Abbott, grants from Regeneron, other from Svelte, grants and other from PhaseBio, grants from Idorsia, grants from Synaptic, personal fees from TobeSoft, grants, personal fees and other from Boehringer Ingelheim, personal fees from Bayer, grants and other from Novo Nordisk, grants from Fractyl, personal fees from Medtelligence/ReachMD, personal fees from CSL Behring, other from Cereno Scientific, grants from Afimmune, personal fees from Ferring Pharmaceuticals, other from CSI, grants from Lexicon, personal fees from MJH Life Sciences, personal fees from Level Ex, personal fees from Contego Medical, personal fees from CellProthera, personal fees from K2P, personal fees from Canadian Medical and Surgical Knowledge Translation Research Group, grants and other from MyoKardia/BMS, grants from Owkin, grants from HLS Therapeutics, grants from Janssen, outside the submitted work. All other authors have no disclosures.

## Appendix Supplementary materials

Image, application 1
