# Supplementary material for: SGLT2 inhibitors decrease cardiovascular death and heart failure hospitalizations in patients with heart failure: A systematic review and meta-analysis
Source: eClinicalMedicine. 2021 Jun 5;36:100933. doi: 10.1016/j.eclinm.2021.100933 (PMC8257984; doi:10.1016/j.eclinm.2021.100933)
Supplement: Supplementary file 1 [file mmc1.docx]

**Search strategy**

PubMed search, January 21, 2021:

(heart failure) AND (SGLT2 OR "sodium-glucose co-transporter-2" OR canagliflozin OR dapagliflozin OR empagliflozin OR sotagliflozin OR ertugliflozin)

1,002 results

**Supplementary figure 1.** Urgent visits for HF were significantly lower in the SGLT2 inhibitor group.


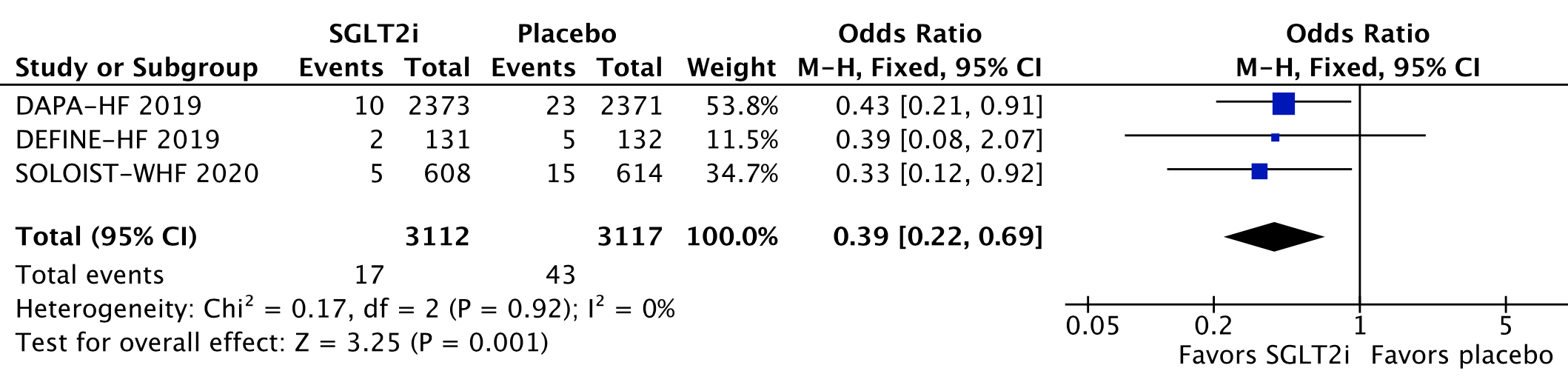


There was a significantly lower incidence of HF-related urgent visits in those treated with SGLT2 inhibitors (HR 0·39; 95% CI 0·22-0·69).

**Supplementary figure 2A.** There was no significant difference between groups in amputations.


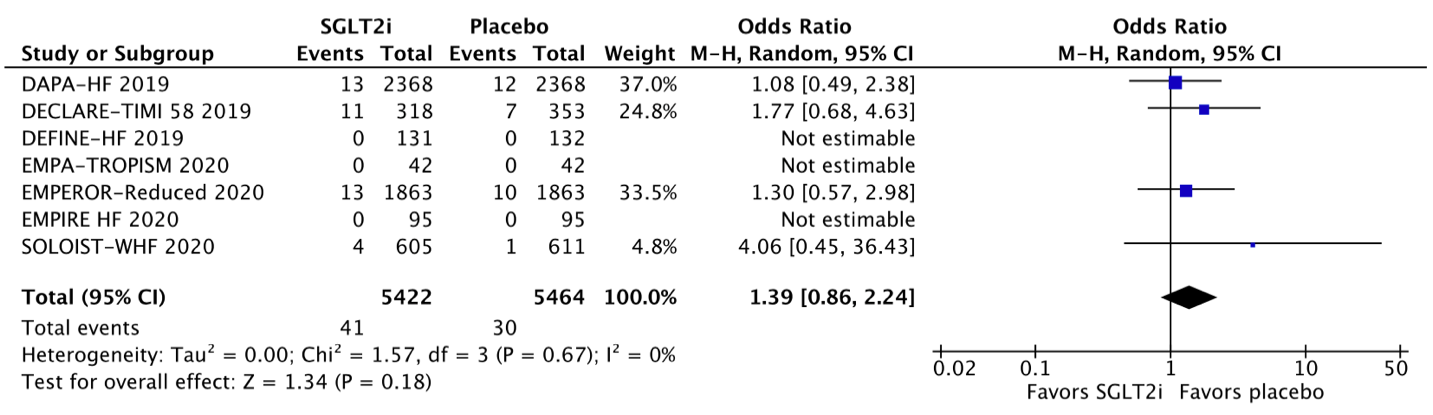


Patients treated with SGLT2 inhibitors (0·75%) and placebo (0·55%) had a similar incidence of amputations (HR 1·39; 95% CI 0·86-2·24).

**Supplementary figure 2B.** There was no significant difference between groups in bone fractures.

**
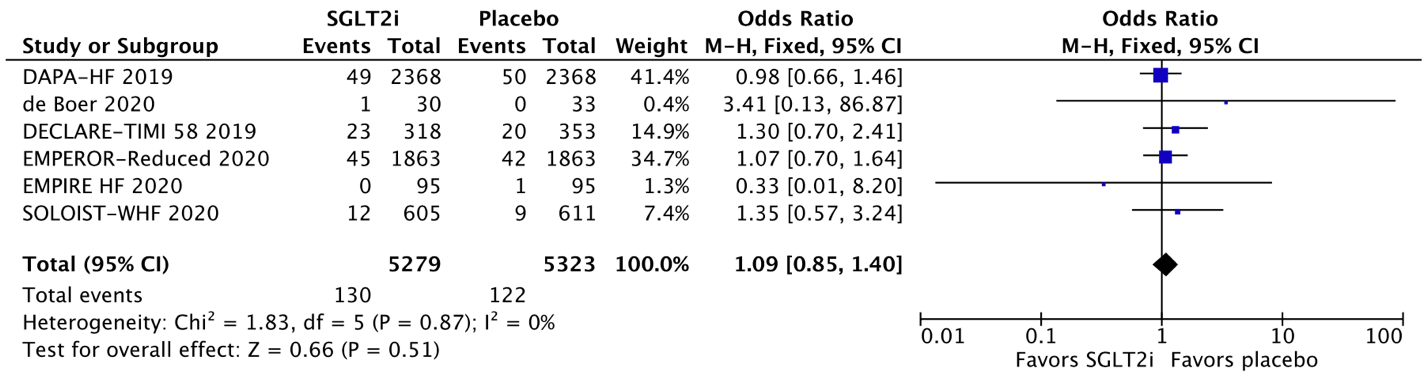
**

Patients treated with SGLT2 inhibitors (2·5%) and placebo (2·3%) had a similar incidence of amputations (HR 1·09; 95% CI 0·85-1·40).

**Supplementary figure 3.** Weight loss was significantly higher in the SGLT2 inhibitor group.

**
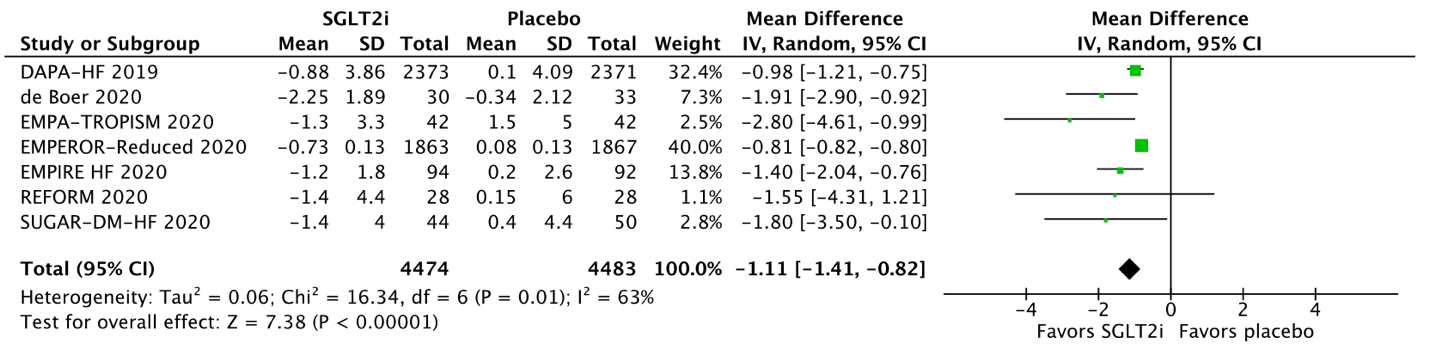
**

Patients treated with SGLT2 inhibitors had a significantly higher mean weight loss compared with those treated with placebo. The mean difference between groups was 1.11 Kg (95% CI 0.82-1.41 Kg).

**Supplementary figure 4A.** Cardiovascular death or HF hospitalizations/urgent visits in men.


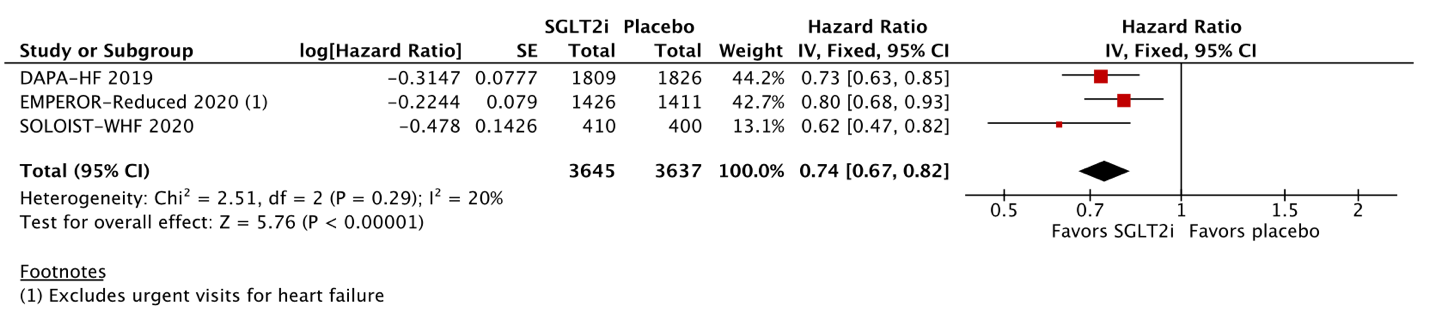
In men with HF, there was a significant 26% relative risk reduction in the composite endpoint of cardiovascular death or HF hospitalizations/urgent visits among those treated with SGLT2 inhibitors compared with placebo (OR 0·74; 95% CI 0·67-0·82).

**Supplementary figure 4B.** Cardiovascular death or HF hospitalizations/urgent visits in women.


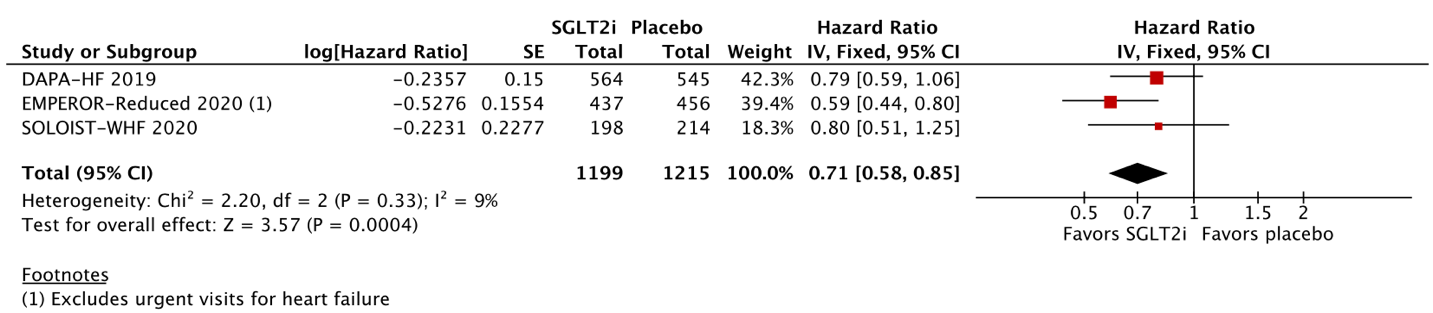
In women with HF, there was a significant 29% relative risk reduction in the composite endpoint of cardiovascular death or HF hospitalizations/urgent visits among those treated with SGLT2 inhibitors compared with placebo (OR 0·71; 95% CI 0·58-0·85).

**Supplementary figure 5A.** Cardiovascular death or HF hospitalizations/urgent visits in patients < 65 years old.


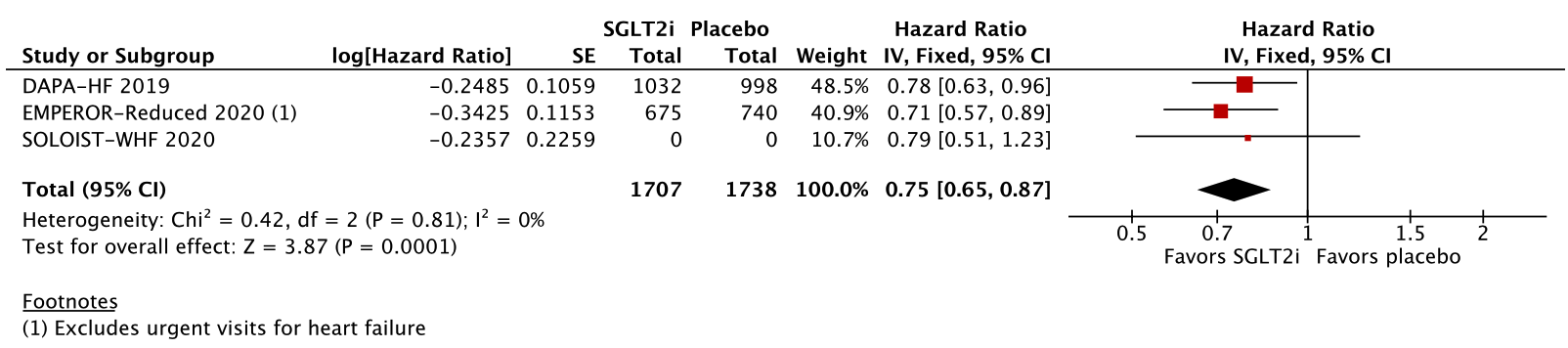


In patients with HF under the age of 65 years, there was a significant 25% relative risk reduction in the composite endpoint of cardiovascular death or HF hospitalizations/urgent visits among those treated with SGLT2 inhibitors compared with placebo (OR 0·75; 95% CI 0·65-0·87). The number of patients < 65 years old stratified between the SGLT2 inhibitor and placebo groups was not available in the SOLOIST-WHF trial.

**Supplementary figure 5B.** Cardiovascular death or HF hospitalizations/urgent visits in patients ≥ 65 years old.

**
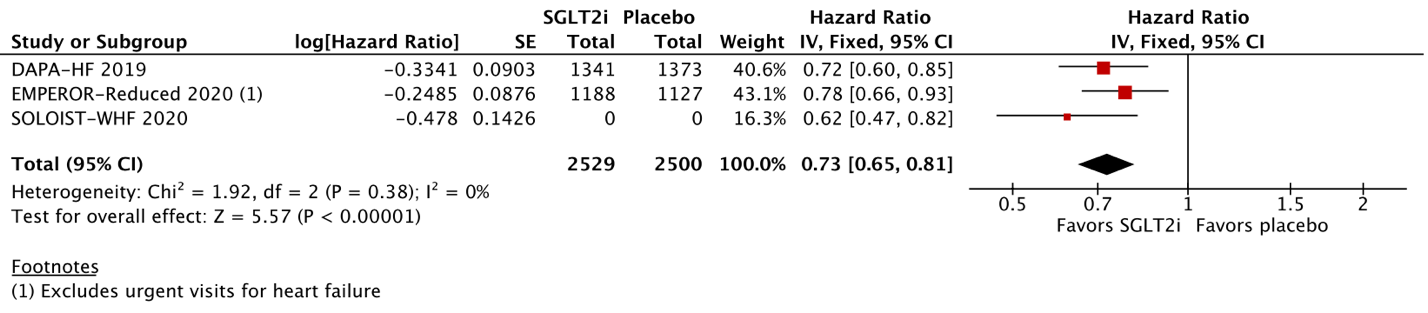
**In patients with HF aged 65 years or older, there was a significant 27% relative risk reduction in the composite endpoint of cardiovascular death or HF hospitalizations/urgent visits among those treated with SGLT2 inhibitors compared with placebo (OR 0·73; 95% CI 0·65-0·81). The number of patients ≥ 65 years old stratified between the SGLT2 inhibitor and placebo groups was not available in the SOLOIST-WHF trial.

**Supplementary figure 6A.** Cardiovascular death or HF hospitalizations/urgent visits in patients with an estimated glomerular filtration rate (eGFR) <60 mL/min/1.73m^2^.


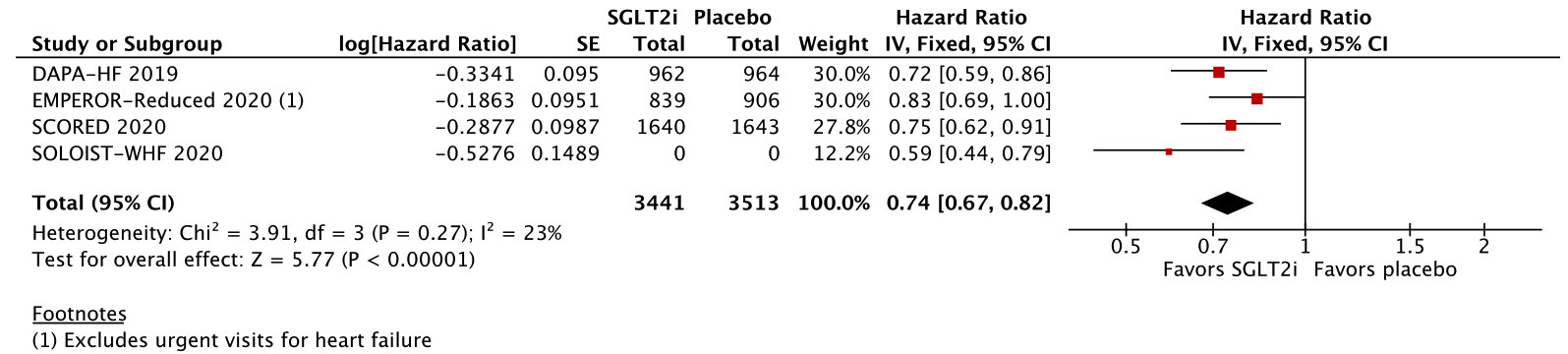


In patients with an eGFR < 60 mL/min/1.73m^2^, there was a significant 26% relative risk reduction in the composite endpoint of cardiovascular death or HF hospitalizations/urgent visits among those treated with SGLT2 inhibitors compared with placebo (OR 0·74; 95% CI 0·67-0·82). The number of patients with eGFR < 60 mL/min/1.73m^2^ stratified between the SGLT2 inhibitor and placebo groups was not available in the SOLOIST-WHF trial.

**Supplementary figure 6B.** Among patients with eGFR ≥60 mL/min/1.73m^2^, cardiovascular death or HF hospitalizations/urgent visits was significantly lower in the SGLT2 inhibitor group.

**
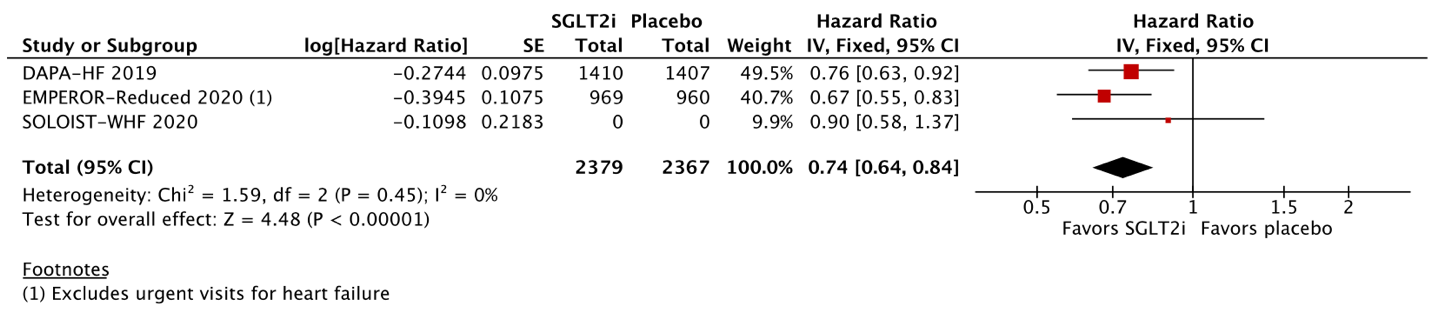
**In patients with an eGFR ≥ 60 mL/min/1.73m^2^, there was a significant 26% relative risk reduction in the composite endpoint of cardiovascular death or HF hospitalizations/urgent visits among those treated with SGLT2 inhibitors compared with placebo (OR 0·74; 95% CI 0·64-0·84). The number of patients with eGFR ≥ 60 mL/min/1.73m^2^ stratified between the SGLT2 inhibitor and placebo groups was not available in the SOLOIST-WHF trial.

**Supplementary figure 7A.** Cardiovascular death or HF hospitalizations/urgent visits in patients with New York Heart Association (NYHA) class II symptoms.


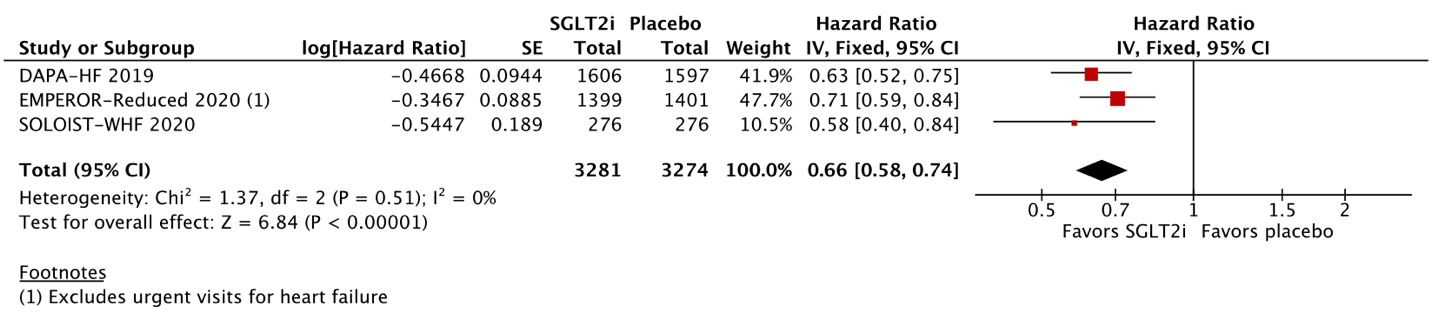


In patients with NYHA class II symptoms, there was a significant 34% relative risk reduction in the composite endpoint of cardiovascular death or HF hospitalizations/urgent visits among those treated with SGLT2 inhibitors compared with placebo (OR 0·66; 95% CI 0·58-0·74).

**Supplementary figure 7B.** Cardiovascular death or HF hospitalizations/urgent visits in patients with New York Heart Association (NYHA) class III or IV symptoms.


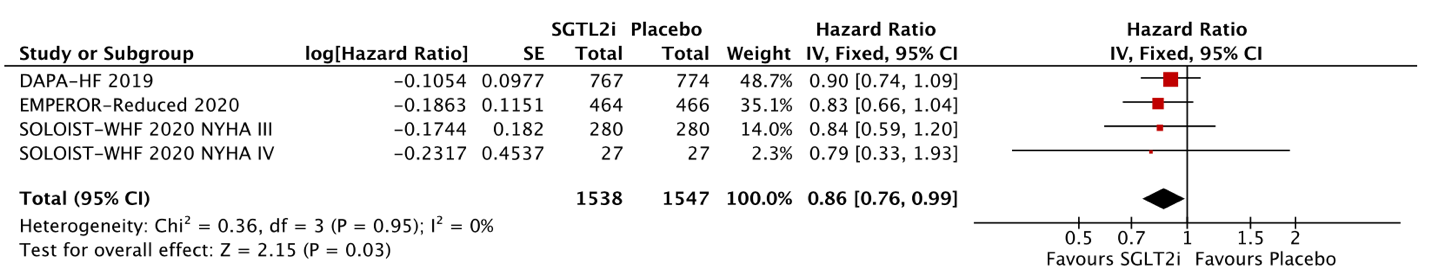


In patients with NYHA class III or IV symptoms, there was a significant 14% relative risk reduction in the composite endpoint of cardiovascular death or HF hospitalizations/urgent visits among those treated with SGLT2 inhibitors compared with placebo (OR 0·86; 95% CI 0·76-0·99).

**Supplementary figure 8A.** Cardiovascular death or HF hospitalizations/urgent visits in patients with diabetes.


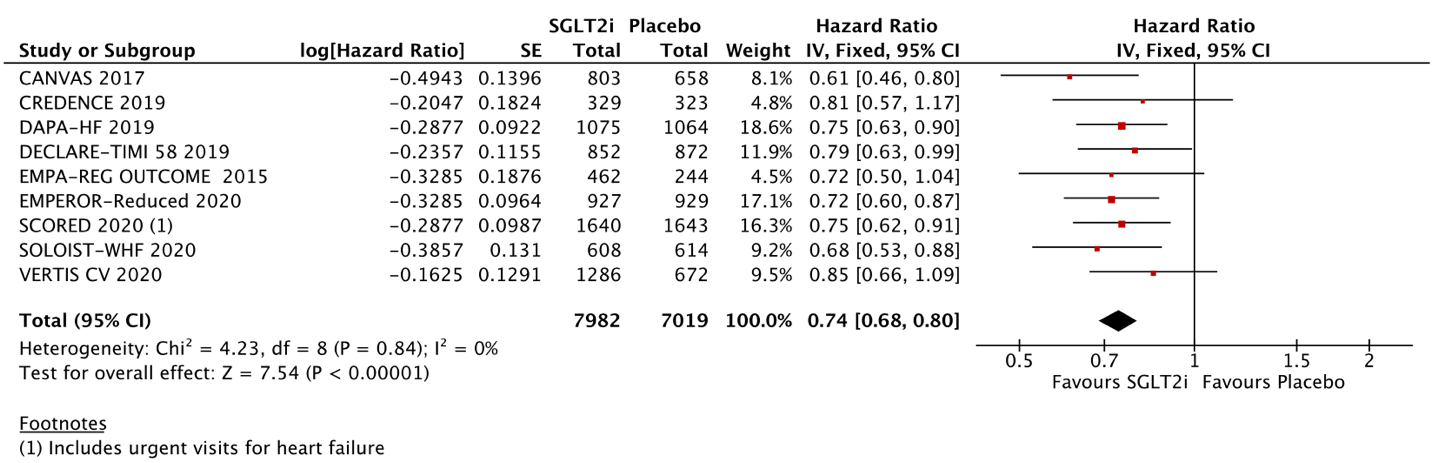


In patients with HF and diabetes, there was a significant 26% relative risk reduction in the composite endpoint of cardiovascular death or HF hospitalizations/urgent visits among those treated with SGLT2 inhibitors compared with placebo (OR 0·74; 95% CI 0·68-0·80).

**Supplementary figure 8B.** Cardiovascular death or HF hospitalizations/urgent visits in patients without diabetes.


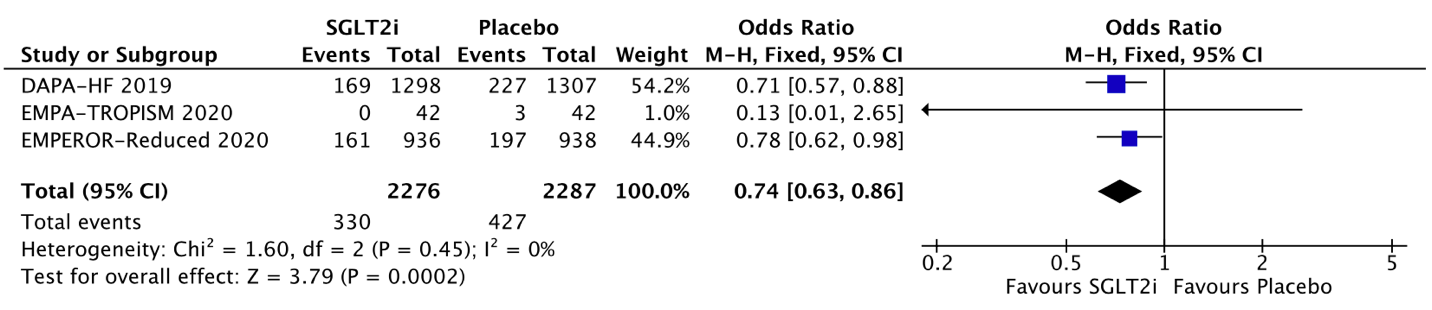


In patients with HF and no diabetes, there was a significant 26% relative risk reduction in the composite endpoint of cardiovascular death or HF hospitalizations/urgent visits among those treated with SGLT2 inhibitors compared with placebo (OR 0·74; 95% CI 0·63-0·86).

**Supplementary figure 9A.** Funnel plot for all-cause mortality


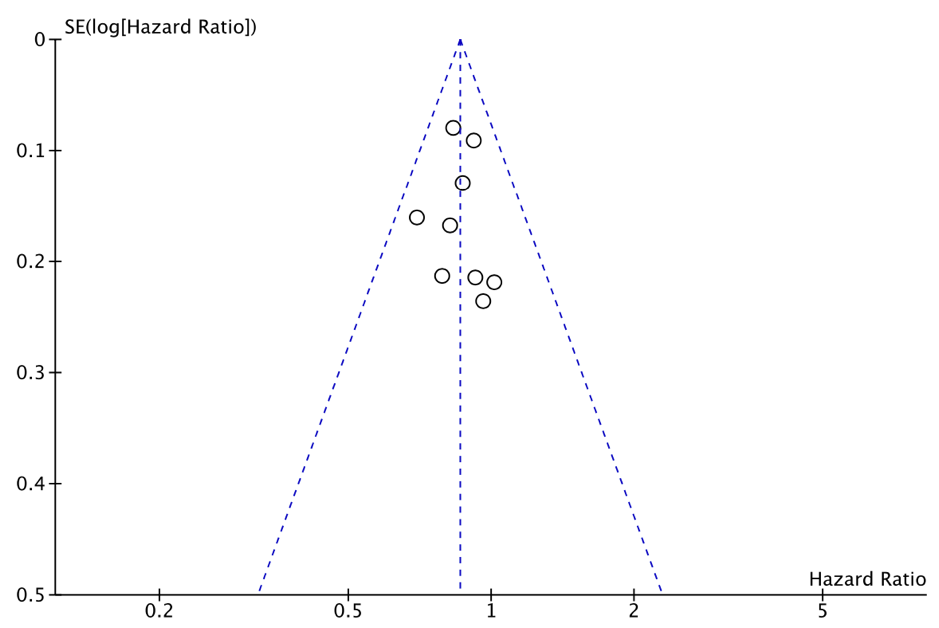


A symmetrical distribution of study weights and hazards ratios shows no evidence of publication bias.

**Supplementary figure 9B.** Funnel plot for cardiovascular mortality


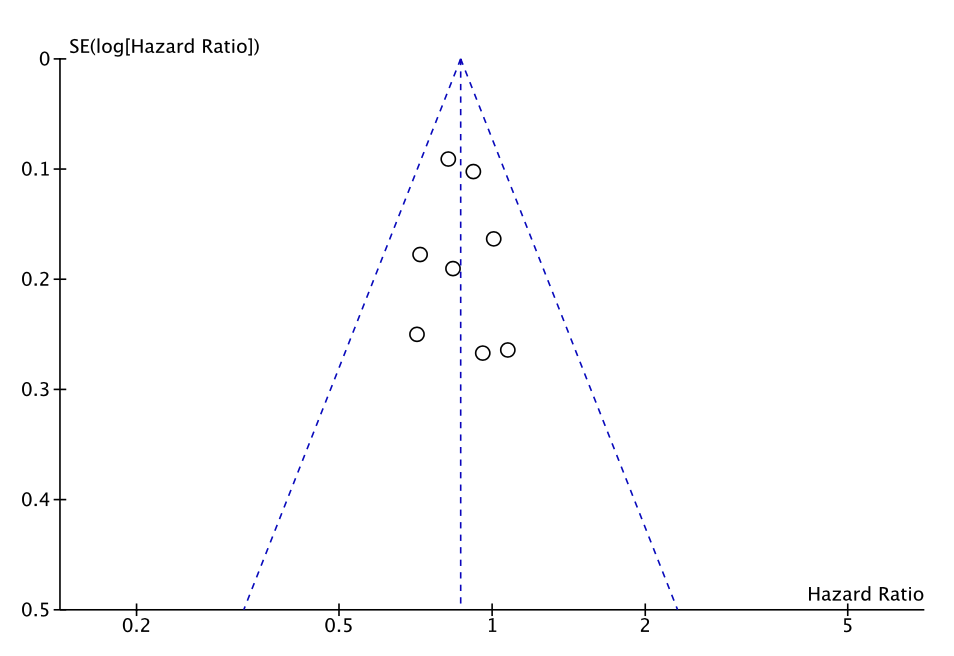


A symmetrical distribution of study weights and hazards ratios shows no evidence of publication bias

**Supplementary Table 1.** Critical appraisal according to the Cochrane Collaboration’s tool for assessing risk of bias in randomized trials.

| **Study** | **Selection bias** | **Performance bias** | **Detection bias** | **Attrition bias** | **Reporting bias** |
| --- | --- | --- | --- | --- | --- |
| De Boer 2020 | Low | Low | Unclear | High | Low |
| CANVAS HF 2018 | Low | Low | Low | Low | Low |
| CREDENCE 2019 | Low | Low | Low | Low | Low |
| DAPA-HF 2019 | Low | Low | Low | Low | Low |
| DECLARE TIMI 2019 | Low | Low | Low | Low | Low |
| DEFINE-HF 2019 | Low | Low | Low | Low | Low |
| EMPA TROPISM 2020 | Low | Low | Low | Low | Low |
| EMPA-REG 2016 | Low | Low | Low | Low | Low |
| EMPEROR-Reduced 2020 | Low | Low | Low | Low | Low |
| REFORM 2020 | Low | Low | Low | Low | Low |
| SCORED 2020 | Low | Low | Low | Low | Low |
| SOLOIST-WHF 2020 | Low | Low | Low | Low | Low |
| VERTIS 2020 | Low | Low | Low | Low | Low |
